# Supplementary material for: A novel platform for attenuating immune hyperactivity using EXO‐CD24 in COVID‐19 and beyond
Source: EMBO Mol Med. 2022 Jul 13;14(9):e15997. doi: 10.15252/emmm.202215997 (PMC9349550; doi:10.15252/emmm.202215997)
Supplement: Supplementary file 1 — Appendix [file EMMM-14-e15997-s002.pdf]

Appendix Supplementary Methods

EXO-CD24 repeated-dose toxicity study in mice, 7 days follow up	
**Table**	**Page**
Animal weight (main group)	2
Animal weight (recovery group)	3
Food consumption (main group)	3
Food consumption (recovery group)	3
Organ weight (main group)	4
Organ weight (recovery group)	5
Urine Analysis (main group)	5
Urine Analysis (recovery group)	6
Clinical Pathology:<br>• Hematology (main group)<br>• Hematology (recovery group)<br>• Clinical chemistry (main group)	7
Clinical chemistry (recovery group)	8

EXO-CD24 repeated-dose toxicity study in mice, 7 days follow up.

1. *Animal Weight (gram)*

Animal weight, main groups:

group	recovery	mouse	weight	weight	weight	weight	weight	weight	weight
Vehicle		8951	18.5	18.3	18.1	18	18.4	18.3	18.1
		8952	19	18.8	18.7	18.7	19.1	19.1	19.2
		8953	19.3	18.7	19.2	19	18.8	18.7	18.8
		8954	18.5	18.1	18.3	18.2	18.3	18.1	18.5
		8955	19.3	19.7	19.4	19.2	19.2	19.5	19.4
	AVERAGE		18.92	18.72	18.74	18.62	18.76	18.74	18.8
	SEM		0.18	0.276405	0.2502	0.22891	0.180555	0.256125	0.234521
Mid dose		8959	17.4	17.6	17.6	18.3	18.1	18.5	18.4
		8960	19.8	19.7	19.5	19.3	19.3	19	19.1
		8961	20	20.1	20.6	20.6	20.5	20.3	20.6
		8962	19.7	19.7	19.8	19.9	20	20.1	19.8
		8963	19.4	19.6	20.4	20.7	20.6	20.6	20.9
		8964	17.8	18.1	17.9	18.2	18.5	18.8	18.7
		8965	18.8	18.9	19.2	19.2	19.4	19.3	19.5
		8966	18.5	18.9	19	18.9	18.8	18.5	18.8
	AVERAGE		18.925	19.075	19.25	19.3875	19.4	19.3875	19.475
	SEM		0.341609	0.307496	0.380789	0.336175	0.324037	0.295464	0.320574
High dose		8970	19	18.5	18.7	18.8	18.7	18.6	18.7
		8971	20.1	19.7	20.1	19.8	20.1	20.2	19.9
		8972	19	18.7	18.9	18.6	18.6	18.9	19.1
		8973	17	16	16.5	16.7	17.1	17.2	17
		8974	19.4	18.8	19.5	19.5	19.8	20	20.2
		8975	19	19.4	19.3	19.5	19.3	19.1	19.4
		8976	19.3	18.5	18.9	19.2	18.8	18.9	18.8
		8977	19.3	18.4	18.7	19	19.2	18.9	19
	AVERAGE		19.0125	18.5	18.825	18.8875	18.95	18.975	19.0125
	SEM		0.314777	0.392792	0.372132	0.34249	0.323485	0.323899	0.341968

Animal weight, recovery groups:

Group		Mouse #	Day 0	Day 1	Day 2	Day 3	Day 4	Day 5
Vehicle	Recovery	8956	18.9	18.1	18.2	18.5	18.7	18.5
		8957	20.2	19	19.8	19.6	19.6	19.8
		8958	19	19.1	19.4	18.9	19.2	19.4
			19.37	18.73	19.13	19.00	19.17	19.23
			0.34	0.26	0.39	0.26	0.21	0.31
Mid dose	Recovery	8967	18.6	18.4	18.2	18.4	18.3	17.9
		8968	17.5	17	18.2	17.8	18.1	17.9
		8969	20.2	20	20.9	20.6	20.3	20
			18.77	18.47	19.10	18.93	18.90	18.60
			0.64	0.71	0.73	0.69	0.57	0.57
High dose	Recovery	8978	19.2	18.1	18.6	18.2	18.5	18.5
		8979	18.2	17.9	18.1	18.1	18.2	17.8
		8980	20	19.7	19.5	19.5	19.3	19.2
			19.13	18.57	18.73	18.60	18.67	18.50

2. *Food Consumption.*

Food was weighed weekly.

Food consumption, main groups (gram):

Group	No of mice	Day 0	Day 6	Total Gr	per mouse
Vehicle	5	389gr	267	122	24.4
Mid dose	8	402gr	214.8	187.2	23.4
High dose	8	386gr	189.2	196.8	24.6

Food consumption, recovery groups (gram):

Group	No of mice	Day 0	Day 6	Total Gr	Per mouse	Day 13	Total Gr	Per mouse
Vehicle	3	405gr	333.9	71.1	23.7	259.3gr	74.6	24.8
Mid dose	3	375gr	300.7	74.3	24.7	229.2gr	71.5	23.8
High dose	3	392gr	324.1	67.9	22.6	251.2gr	72.9	24.3

3. *Organ Weight.*

After sacrifice, animal organs were isolated and weighed.

Organ weight, main groups (gram).

group	mouse	brain	heart	lungs	thymus	spleen	kidneys	liver
Vehicle	8951	0.429	0.109	0.268	0.051	0.078	0.244	0.813
	8952	0.447	0.122	0.269	0.042	0.086	0.252	0.872
	8953	0.449	0.091	0.397	0.051	0.083	0.247	0.789
	8954	0.448	0.095	0.276	0.047	0.091	0.24	0.759
	8955	0.458	0.104	0.285	0.051	0.093	0.28	0.913
	AVERAGE	0.4462	0.1042	0.299	0.0484	0.0862	0.2526	0.8292
	SEM	0.004228	0.004894	0.022082	0.00159	0.002423	0.006372	0.025029
Mid dose	8959	0.429	0.089	0.279	0.058	0.063	0.212	0.737
	8960	0.43	0.085	0.395	0.034	0.063	0.245	0.78
	8961	0.435	0.101	0.249	0.044	0.071	0.27	0.779
	8962	0.433	0.1	0.315	0.183	0.444	0.301	1.184
	8963	0.417	0.099	0.318	0.035	0.07	0.229	0.733
	8964	0.414	0.096	0.272	0.048	0.087	0.222	0.81
	8965	0.437	0.094	0.388	0.039	0.068	0.25	0.718
	8966	0.414	0.11	0.352	0.038	0.069	0.251	0.696
	AVERAGE	0.426125	0.09675	0.321	0.059875	0.116875	0.2475	0.804625
	SEM	0.003393	0.002724	0.019049	0.017808	0.046807	0.010041	0.055778
High dose	8970	0.406	0.086	0.386	0.036	0.074	0.285	0.762
	8971	0.449	0.102	0.357	0.041	0.094	0.273	0.82
	8972	0.423	0.09	0.32	0.033	0.086	0.26	0.745
	8973	0.433	0.107	0.371	0.042	0.078	0.245	0.678
	8974	0.403	0.101	0.348	0.035	0.075	0.282	0.763
	8975	0.406	0.108	0.336	0.053	0.072	0.254	0.682
	8976	0.446	0.107	0.321	0.07	0.077	0.252	0.766
	8977	0.394	0.115	0.338	0.038	0.081	0.249	0.792
	AVERAGE	0.42	0.102	0.347125	0.0435	0.079625	0.2625	0.751
	SEM	0.007382	0.003423	0.008243	0.004371	0.00257	0.005467	0.017453

Organ weight, recovery groups (gram)

Group	Mouse #	Brain	Hear	Lungs	Thymus	Spleen	Kidneys	Liver
Vehicle	8956	0.445	0.126	0.308	0.034	0.085	0.3	0.994
	8957	0.448	0.124	0.271	0.041	0.097	0.238	0.982
	8958	0.444	0.109	0.357	0.046	0.103	0.289	1.143
	AVERAGE	0.446	0.120	0.312	0.040	0.095	0.276	1.040
	SEM	0.001	0.004	0.020	0.003	0.004	0.016	0.042
Mid dose	8967	0.439	0.095	0.274	0.05	0.097	0.241	0.864
	8968	0.425	0.092	0.259	0.041	0.084	0.224	0.779
	8969	0.442	0.093	0.229	0.046	0.086	0.257	1.03
	AVERAGE	0.435	0.093	0.254	0.046	0.089	0.241	0.891
	SEM	0.004	0.001	0.011	0.002	0.003	0.008	0.060
High dose	8978	0.447	0.102	0.26	0.051	0.092	0.247	0.9
	8979	0.44	0.085	0.277	0.051	0.087	0.255	0.802
	8980	0.45	0.1	0.287	0.049	0.1	0.265	0.977
	AVERAGE	0.446	0.096	0.275	0.050	0.093	0.256	0.893
	SEM	0.002	0.004	0.006	0.001	0.003	0.004	0.041

4. Urine Analysis

On termination day, urine was collected and analyzed.

Urine analysis results, main groups:

group	mouse	Leukocytes	Urobilinogen	Bilirubin (mg/dL)	Blood	Nitrite	pH	Specific Gravity	Protein(mg/dL)	Glucose	Keytone
Vehicle	8951	negative	Normal	0.5	negative	negative	5	1.03	30	negative	negative
	8952	negative	Normal	0.5	negative	negative	6	1.03	30	negative	negative
	8953	negative	Normal	0.5	negative	negative	6	1.03	30	negative	negative
	8954	negative	Normal	0.5	negative	negative	5	1.03	30	negative	negative
	8955	negative	Normal	0.5	negative	negative	6	1.03	30	negative	negative
Mid dose	8959	negative	Normal	0.5	negative	negative	6	1.03	30	negative	negative
	8960	negative	Normal	0.5	negative	negative	5	1.03	30	negative	negative
	8961	negative	Normal	0.5	negative	negative	6	1.03	30	negative	negative
	8962	negative	Normal	0.5	negative	negative	6	1.03	30	negative	negative
	8963	negative	Normal	0.5	negative	negative	6	1.03	30	negative	negative
	8964	negative	Normal	0.5	negative	negative	6	1.03	30	negative	negative
	8965	negative	Normal	0.5	negative	negative	6	1.03	30	negative	negative
	8966	negative	Normal	0.5	negative	negative	5	1.03	30	negative	negative
High dose	8970	negative	Normal	0.5	negative	negative	5	1.03	30	negative	negative
	8971	negative	Normal	0.5	negative	negative	6	1.03	30	negative	negative
	8972	negative	Normal	0.5	negative	negative	6	1.03	30	negative	negative
	8973	negative	Normal	0.5	negative	negative	5	1.03	30	negative	negative
	8974	negative	Normal	0.5	negative	negative	5	1.03	30	negative	negative
	8975	negative	Normal	0.5	negative	negative	6	1.03	30	negative	negative
	8976	negative	Normal	0.5	negative	negative	6	1.03	30	negative	negative
	8977	negative	Normal	0.5	negative	negative	6	1.03	30	negative	negative

Urine analysis results, recovery groups

Group	mouse	Leukocytes	Urobilinogen	Bilirubin (mg/dL)	Blood	Nitrite	pH	Specific Gravity
Vehicle	8956	Negative	Normal	0.5	Negative	Negative	6	1.03
	8957	Negative	Normal	0.5	Negative	Negative	5	1.03
	8958	Negative	Normal	0.5	Negative	Negative	6	1.03
Mid dose	8967	Negative	Normal	0.5	Negative	Negative	5	1.03
	8968	Negative	Normal	0.5	Negative	Negative	5	1.03
	8969	Negative	Normal	0.5	Negative	Negative	6	1.03
High dose	8978	Negative	Normal	0.5	Negative	Negative	6	1.03
	8979	Negative	Normal	0.5	Negative	Negative	5	1.03
	8980	Negative	Normal	0.5	Negative	Negative	5	1.03

Urine analysis results, recovery groups (continue):

Group	mouse	Protein (mg/dL)	Glucose	Ketone
Vehicle	8956	30	Negative	Negative
	8957	30	Negative	Negative
	8958	30	Negative	Negative
Mid dose	8967	30	Negative	Negative
	8968	30	Negative	Negative
	8969	30	Negative	Negative
High dose	8978	30	Negative	Negative
	8979	30	Negative	Negative
	8980	30	Negative	Negative

5. *Clinical Pathology*

At the end of the study, blood was collected and analyzed.

Hematology results, main groups:

		3.2-24.3	7.0-11.0	11.8-18.0	36.7-59.5	43.5-67	15.8-21.20	31.4-36	0.5-8.8		3.0-20.6	0.0-0.7	0.0-1.	0.0-0.0	674.0–16	0.5-8.8		3.0-20.6	0.0-0.7	0.0-1.0	0.0-0.0
	Animal	WBC	RBC	HGB	HCT	MCV	MCH	MCHC	Neut	Bands	Lymph	Mono	Eos	Baso	Plate	Neutr Abs	Bands Abs	Lymph Abs	Mono Abs	Eos Abs	Basos Abs
group	ID	10*3/µl	10*6/µl	g/dl	%	FL	pg	g/dl	%	%	%	%	%	%	10*3/µl	10*3/µl	10*3/µl	10*3/µl	10*3/µl	10*3/µl	10*3/µl
Vehicle	8951	7.01	11.69	17.5	51	43.6	15	34.3	22	0	76	2	0	0	973	1.54	0	5.33	0.14	0	0
	8952	7.86	11.51	17.4	52.1	45.3	15.1	33.4	14	0	86	0	0	0	863	1.1	0	6.76	0	0	0
	8953	7.63	12.04	18.1	53.7	44.6	15	33.7	32	0	66	2	0	0	1003	2.44	0	5.04	0.15	0	0
	8954	15.04	11.05	16.7	53.7	48.6	15.1	31.1	30	0	68	2	0	0	996	4.51	0	10.23	0.3	0	0
	8955	10.62	10.96	16.2	51.5	47	14.8	31.5	24	0	74	2	0	0	1063	2.55	0	7.86	0.21	0	0
	AVERAGE	9.63	11.45	17.18	52.40	45.82	15.00	32.80	24.40	0.00	74.00	1.60	0.00	0.00	979.60	2.43	0.00	7.04	0.16	0.00	0.00
	SEM	1.49	0.20	0.33	0.56	0.89	0.05	0.63	3.19	0.00	3.52	0.40	0.00	0.00	32.72	0.59	0.00	0.94	0.05	0.00	0.00
Mid dose	8959	8.15	10.98	16.5	49.6	45.2	15	33.3	24	0	74	2	0	0	1035	1.96	0	6.03	0.16	0	0
	8960	4.02	11.51	17.6	50.1	43.5	15.3	35.1	24	0	72	2	2	0	1136	0.96	0	2.89	0.08	0.08	0
	8961	7.13	11.79	17.7	55.7	47.2	15	31.8	22	0	74	2	2	0	1074	1.57	0	5.28	0.14	0.14	0
	8962	10.19	10.61	15.4	47.6	44.9	14.5	32.4	20	0	76	4	0	0	391	2.04	0	7.74	0.41	0	0
	8963	9.96	11.88	17.8	50.5	42.5	15	35.2	16	0	78	4	2	0	1075	1.59	0	7.77	0.4	0.2	0
	8964	7.32	11.24	16.9	50.3	44.8	15	33.6	26	0	68	2	4	0	1057	1.9	0	4.98	0.15	0.29	0
	8965	9.68	12.22	18.2	51.8	42.4	14.9	35.1	30	0	70	0	0	0	1000	2.9	0	6.78	0	0	0
	8966	8.59	11.13	16.9	48.7	43.8	15.2	34.7	24	0	76	0	0	0	943	2.06	0	6.53	0	0	0
	AVERAGE	8.13	11.42	17.13	50.54	44.29	14.99	33.90	23.25	0.00	73.50	2.00	1.25	0.00	963.88	1.87	0.00	6.00	0.17	0.09	0.00
	SEM	0.72	0.19	0.32	0.86	0.56	0.08	0.47	1.46	0.00	1.18	0.53	0.53	0.00	84.28	0.20	0.00	0.57	0.06	0.04	0.00
High dose	8970	15.4	11.33	17.3	48.6	42.9	15.3	35.6	14	0	48	36	2	0	1012	2.16	0	7.39	5.54	0.31	0
	8971	9.83	10.94	16.6	47.5	43.4	15.2	34.9	24	0	76	0	0	0	865	2.36	0	7.47	0	0	0
	8972	13.03	11.54	17.7	50.4	43.7	15.3	35.1	28	0	71	1	0	0	963	3.65	0	9.25	0.13	0	0
	8973	9.42	10.91	16.6	47.5	43.5	15.2	34.9	16	0	82	2	0	0	900	1.51	0	7.72	0.19	0	0
	8974	10.14	11.45	17.1	49.2	43	14.9	34.8	16	0	80	4	0	0	993	1.62	0	8.11	0.41	0	0
	8975	6.26	10.95	16.7	47.5	43.4	15.3	35.2	24	0	76	0	0	0	875	1.5	0	4.76	0	0	0
	8976	8.11	10.9	16.6	47	43.1	15.2	35.3	26	0	70	4	0	0	907	2.11	0	5.68	0.32	0	0
	8977	10.64	11.09	16.9	49	44.2	15.2	34.5	28	0	68	2	2	0	934	2.98	0	7.24	0.21	0.21	0
	AVERAGE	10.35	11.14	16.94	48.34	43.40	15.20	35.04	22.00	0.00	71.38	6.13	0.50	0.00	931.13	2.24	0.00	7.20	0.85	0.07	0.00
	SEM	1.00	0.09	0.14	0.41	0.15	0.05	0.12	2.04	0.00	3.76	4.30	0.33	0.00	19.12	0.27	0.00	0.49	0.67	0.04	0.00

Hematology results, recovery groups:

		Neut	Bands	Lymph	Mono	Eos	Baso	Plate
		%	%	%	%	%	%	10*3/µl
Group	Animal No. /Reference	0.5-8.8		3.0-20.6	0.0-0.7	0.0-1.0	0.0-0.0	674.0–1675.0
Vehicle	8956	10.00	0.00	86.00	2.00	2.00	0.00	814.00
	8957	24.00	0.00	70.00	2.00	4.00	0.00	683.00
	8958	30.00	0.00	68.00	0.00	2.00	0.00	806.00
	AVERAGE	21.33	0.00	74.67	1.33	2.67	0.00	767.67
	SEM	4.84	0.00	4.65	0.54	0.54	0.00	34.62
Mid dose	8967	24.00	0.00	74.00	2.00	0.00	0.00	706.00
	8968	26.00	0.00	68.00	2.00	4.00	0.00	828.00
	8969	14.00	0.00	84.00	2.00	0.00	0.00	733.00
	AVERAGE	21.33	0.00	75.33	2.00	1.33	0.00	755.67
	SEM	3.03	0.00	3.81	0.00	1.09	0.00	30.21
High dose	8978	36.00	0.00	58.00	4.00	2.00	0.00	681.00
	8979	32.00	0.00	66.00	2.00	0.00	0.00	674.00
	8980	28.00	0.00	62.00	2.00	8.00	0.00	672.00
	AVERAGE	32.00	0.00	62.00	2.67	3.33	0.00	675.67
	SEM	1.89	0.00	1.89	0.54	1.96	0.00	2.23

		Neutr Abs	Bands Abs	Lymph Abs	Mono Abs	Eos Abs	Basos Abs
		10*3/µl	10*3/µl	10*3/µl	10*3/µl	10*3/µl	10*3/µl
Group	Animal No. /Reference	0.5-8.8		3.0-20.6	0.0-0.7	0.0-1.0	0.0-0.0
Vehicle	8956	0.79	0.00	6.79	0.16	0.16	0
	8957	2.03	0.00	5.93	0.17	0.34	0
	8958	3.31	0.00	7.50	0.00	0.22	0
	AVERAGE	2.04	0.00	6.74	0.11	0.24	0.00
	SEM	0.59	0.00	0.37	0.04	0.04	0.00
Mid Dose	8967	1.98	0.00	6.11	0.17	0.00	0
	8968	1.80	0.00	4.71	0.14	0.28	0
	8969	0.83	0.00	5.00	0.12	0.00	0
	AVERAGE	1.54	0.00	5.27	0.14	0.09	0.00
	SEM	0.29	0.00	0.35	0.01	0.08	0.00
High Dose	8978	2.42	0.00	3.90	0.27	0.13	0
	8979	1.73	0.00	3.58	0.11	0.00	0
	8980	2.78	0.00	6.16	0.20	0.80	0
	AVERAGE	2.31	0.00	4.55	0.19	0.31	0.00
	SEM	0.25	0.00	0.66	0.04	0.20	0.00

Clinical chemistry results, main groups:

		0.1-0.4	8.9-14.5	3.5-19.8	46.0-282.	18.0-103.	51.0-239.	1.0-6.8	2.0-4.9	1.3-3.6		0.1-0.3	29.0-356.0	255-1879	36.0-361.	15.0-296.0	15.00-309.01		149.0-167.	4.9-11.2	95-115	
	Animal	Creat	Calc	Phos	Gluc	Urea	Chol	TP	Alb	Glob	Alb/Glob	T. Bil	Alk Phos	LDH	SGOT	SGPT	Trig	CPK	Na	K	Cl	GGTP
group	ID	mg/dl	mg/dl	mg/dl	mg/dl	mg/dl	mg/dl	g/dl	g/dl	g/dl	Ratio	mg/dl	IU/L	IU/L	IU/L	IU/L	mg/dl	IU/L	mmol/L	mmol/L	mmol/L	IU/L
Vehicle	8951.00	0.17	10.13	9.50	149.00	34.80	101.00	5.30	3.80	1.50	2.53	0.08	221.00	1108.00	171.00	47.00	87.00	1996.00	152.00	7.40	113.00	0.00
	8952.00	0.17	9.72	10.00	110.00	28.00	111.00	5.22	3.70	1.52	2.43	0.11	210.00	1120.00	151.00	37.00	70.00	1482.00	152.00	6.40	113.00	0.00
	8953.00	0.18	10.46	10.50	124.00	35.10	116.00	5.59	3.90	1.69	2.31	0.09	200.00	966.00	186.00	43.00	69.00	1958.00	153.00	8.70	110.00	0.00
	8954.00	0.17	10.38	11.60	80.00	30.00	108.00	5.56	3.60	1.96	1.84	0.08	218.00	656.00	182.00	34.00	102.00	1488.00	150.00	9.40	105.00	0.00
	8955.00	0.17	9.97	10.10	114.00	36.70	102.00	5.36	3.90	1.46	2.67	0.08	188.00	841.00	91.00	31.00	132.00	774.00	151.00	7.80	112.00	0.00
	AVERAGE	0.17	10.13	10.34	115.40	32.92	107.60	5.41	3.78	1.63	2.36	0.09	207.40	938.20	156.20	38.40	92.00	1539.60	151.60	7.94	110.60	0.00
	SEM	0.00	0.14	0.35	11.15	1.66	2.80	0.07	0.06	0.09	0.14	0.01	6.06	87.10	17.39	2.93	11.70	220.85	0.51	0.52	1.50	0.00
Mid dose	8959.00	0.17	10.36	10.80	96.00	27.20	110.00	5.82	4.40	1.42	3.10	0.10	200.00	758.00	266.00	54.00	84.00	4190.00	154.00	8.80	108.00	0.00
	8960.00	0.18	9.97	8.10	127.00	28.60	92.00	5.80	3.80	2.00	1.90	0.08	189.00	1195.00	161.00	36.00	64.00	1443.00	152.00	8.00	113.00	0.00
	8961.00	0.17	10.25	9.20	45.00	31.40	91.00	5.77	4.20	1.57	2.68	0.05	220.00	1531.00	200.00	40.00	92.00	2036.00	153.00	7.80	109.00	0.00
	8962.00	0.17	10.27	8.00	121.00	26.50	115.00	7.01	3.20	3.81	0.84	0.07	139.00	1640.00	219.00	45.00	82.00	1867.00	147.00	8.30	109.00	0.00
	8963.00	0.22	10.05	11.60	105.00	26.10	100.00	5.42	4.30	1.12	3.84	0.10	162.00	931.00	123.00	30.00	75.00	1238.00	153.00	7.10	111.00	0.00
	8964.00	0.19	9.77	7.70	157.00	33.00	106.00	5.16	3.80	1.36	2.79	0.09	217.00	1420.00	138.00	36.00	73.00	1231.00	150.00	8.00	113.00	0.00
	8965.00	0.18	10.14	8.80	133.00	34.00	110.00	5.61	4.00	1.61	2.48	0.08	177.00	810.00	810.00	37.00	73.00	1531.00	153.00	7.90	112.00	0.00
	8966.00	0.17	10.51	11.30	99.00	35.90	89.00	5.32	3.80	1.52	2.50	0.11	153.00	1071.00	182.00	35.00	64.00	1890.00	153.00	8.30	114.00	0.00
	AVERAGE	0.18	10.17	9.44	110.38	30.34	101.63	5.74	3.94	1.80	2.52	0.09	182.13	1169.50	262.38	39.13	75.88	1928.25	151.88	8.03	111.13	0.00
	SEM	0.01	0.08	0.56	11.74	1.32	3.55	0.20	0.13	0.30	0.31	0.01	10.49	118.07	79.88	2.61	3.44	340.38	0.81	0.17	0.79	0.00
High dose	8970.00	0.19	9.98	9.40	122.00	36.40	82.00	5.12	3.90	1.22	3.20	0.08	228.00	840.00	117.00	34.00	51.00	903.00	151.00	7.10	113.00	0.00
	8971.00	0.17	9.96	11.90	117.00	26.70	94.00	5.14	3.60	1.54	2.34	0.12	209.00	1144.00	158.00	37.00	59.00	1964.00	153.00	9.60	113.00	0.00
	8972.00	0.18	10.20	9.50	114.00	33.80	94.00	5.43	4.20	1.23	3.41	0.12	185.00	771.00	145.00	37.00	52.00	1452.00	150.00	8.00	109.00	0.00
	8973.00	0.17	9.45	10.80	109.00	26.70	82.00	4.84	3.60	1.24	2.90	0.11	174.00	1027.00	158.00	33.00	54.00	1561.00	150.00	9.10	110.00	0.00
	8974.00	0.21	9.99	10.30	112.00	35.00	90.00	5.45	4.00	1.45	2.76	0.08	195.00	696.00	179.00	32.00	59.00	1711.00	151.00	9.30	112.00	0.00
	8975.00	0.17	9.84	8.60	149.00	37.60	91.00	5.43	3.80	1.63	2.33	0.07	204.00	793.00	205.00	31.00	44.00	2432.00	151.00	9.10	112.00	0.00
	8976.00	0.17	9.60	8.60	160.00	36.30	87.00	5.17	4.10	1.07	3.83	0.08	212.00	656.00	184.00	32.00	63.00	1904.00	150.00	9.20	111.00	0.00
	8977.00	0.17	9.80	8.00	132.00	35.80	96.00	5.66	4.00	1.66	2.41	0.10	218.00	648.00	136.00	44.00	78.00	1038.00	146.00	9.40	106.00	0.00
	AVERAGE	0.18	9.85	9.64	126.88	33.54	89.50	5.28	3.90	1.38	2.90	0.10	203.13	821.88	160.25	35.00	57.50	1620.63	150.25	8.85	110.75	0.00
	SEM	0.01	0.08	0.46	6.60	1.54	1.91	0.09	0.08	0.08	0.19	0.01	6.26	63.13	10.02	1.51	3.59	176.83	0.70	0.30	0.84	0.00

Clinical chemistry results, recovery groups:

		Creat	Calc	Phos	Gluc	Urea	Chol	TP
		mg/dl	mg/dl	mg/dl	mg/dl	mg/dl	mg/dl	g/dl
**group**	**Animal No. /Reference**	**0.1-0.4**	**8.9-14.5**	**3.5-19.8**	**46.0-282.0**	**18.0-103.9**	**51.0-239.0**	**1.0-6.8**
Vehicle	8956	0.21	10.38	8.20	100.00	47.00	120.00	5.59
	8957	0.21	10.44	7.80	144.00	38.50	104.00	5.35
	8958	0.20	10.03	8.90	112.00	40.70	117.00	5.47
	AVERAGE	0.21	10.28	8.30	118.67	42.07	113.67	5.47
	SEM	0.00	0.10	0.26	10.72	2.08	4.01	0.06
Mid dose	8967	0.18	9.59	7.70	129.00	48.00	102.00	5.17
	8968	0.19	9.72	8.20	134.00	55.40	100.00	5.32
	8969	0.18	10.03	7.60	158.00	41.30	105.00	5.16
	AVERAGE	0.18	9.78	7.83	140.33	48.23	102.33	5.22
	SEM	0.00	0.11	0.15	7.31	3.32	1.19	0.04
High dose	8978	0.19	9.85	7.70	150.00	50.80	107.00	5.16
	8979	0.18	10.13	8.40	136.00	32.60	109.00	5.35
	8980	0.26	10.41	8.60	129.00	41.20	119.00	5.53
	AVERAGE	0.21	10.13	8.23	138.33	41.53	111.67	5.35
	SEM	0.02	0.13	0.22	5.04	4.29	3.03	0.09

		SGPT	Trig	CPK	Na	K	Cl	GGTP
		IU/L	mg/dl	IU/L	mmol/L	mmol/L	mmol/L	IU/L
**group**	**Animal No. /Reference**	**15.0-296.0**	15.00-309.01		**149.0-167.0**	**4.9-11.2**	**95-115**	
Vehicle	8956	40.00	204.00	1475.00	154.00	8.10	112.00	0.00
	8957	36.00	238.00	1666.00	152.00	7.60	111.00	0.00
	8958	34.00	161.00	1046.00	153.00	7.50	110.00	0.00
	AVERAGE	36.67	201.00	1395.67	153.00	7.73	111.00	0.00
	SEM	1.44	18.19	149.68	0.47	0.15	0.47	0.00
Mid dose	8967	41.00	228.00	1482.00	151.00	7.50	115.00	0.00
	8968	42.00	227.00	1993.00	152.00	7.80	115.00	0.00
	8969	41.00	202.00	1173.00	151.00	7.90	116.00	0.00
	AVERAGE	41.33	219.00	1549.33	151.33	7.73	115.33	0.00
	SEM	0.27	6.94	195.22	0.27	0.10	0.27	0.00
High dose	8978	38.00	130.00	1380.00	153.00	7.80	114.00	0.00
	8979	45.00	134.00	1592.00	152.00	8.10	114.00	0.00
	8980	40.00	217.00	1901.00	154.00	8.00	113.00	0.00
	AVERAGE	41.00	160.33	1624.33	153.00	7.97	113.67	0.00
	SEM	1.70	23.15	123.51	0.47	0.07	0.27	0.00

		Alb	Glob	Alb/Glob	T. Bil	Phos	LDH	SGOT
		g/dl	g/dl	Ratio	mg/dl	IU/L	IU/L	IU/L
**group**	**Animal No. /Reference**	**2.0-4.9**	**1.3-3.6**		**0.1-0.3**	**29.0-356.0**	**255-1879**	**36.0-361.0**
Vehicle	8956	4.00	1.59	2.53	0.10	251.00	1615.00	134.00
	8957	3.80	1.55	2.33	0.08	220.00	1013.00	144.00
	8958	4.00	1.47	2.74	0.14	200.00	1410.00	128.00
	AVERAGE	3.93	1.54	2.53	0.11	223.67	1346.00	135.33
	SEM	0.05	0.03	0.10	0.01	12.11	144.28	3.81
Mid dose	8967	4.00	1.17	2.62	0.09	220.00	1085.00	138.00
	8968	4.10	1.22	3.02	0.08	227.00	1069.00	171.00
	8969	4.00	1.16	2.46	0.06	201.00	554.00	124.00
	AVERAGE	4.03	1.18	2.70	0.08	216.00	902.67	144.33
	SEM	0.03	0.02	0.14	0.01	6.34	142.39	11.38
High dose	8978	3.90	1.26	2.66	0.09	201.00	886.00	145.00
	8979	4.10	1.25	2.65	0.06	200.00	1026.00	164.00
	8980	4.20	1.33	2.75	0.12	234.00	1303.00	158.00
	AVERAGE	4.07	1.28	2.69	0.09	211.67	1071.67	155.67
	SEM	0.07	0.02	0.03	0.01	9.12	100.04	4.58
